# Long Noncoding RNA: Regulatory Mechanisms and Therapeutic Potential in Sepsis

**DOI:** 10.3389/fcimb.2021.563126

**Published:** 2021-05-12

**Authors:** Wei Wang, Ni Yang, Ri Wen, Chun-Feng Liu, Tie-Ning Zhang

**Affiliations:** Department of Pediatrics, Shengjing Hospital of China Medical University, Shenyang, China

**Keywords:** lncRNA, sepsis, biomarker, inflammation, cardiovascular dysfunction, acute lung injury, acute kidney injury

## Abstract

Sepsis is a life-threatening organ dysfunction caused by a dysregulated host response to infection and is characterized by a hyperinflammatory state accompanied by immunosuppression. Long noncoding RNAs (lncRNAs) are noncoding RNAs longer than 200 nucleotides and have important roles in mediating various biological processes. Recently, lncRNAs were found to exert both promotive and inhibitory immune functions in sepsis, thus participating in sepsis regulation. Additionally, several studies have revealed that lncRNAs are involved in sepsis-induced organ dysfunctions, including cardiovascular dysfunction, acute lung injury, and acute kidney injury. Considering the lack of effective biomarkers for early identification and specific treatment for sepsis, lncRNAs may be promising biomarkers and even targets for sepsis therapies. This review systematically highlights the recent advances regarding the roles of lncRNAs in sepsis and sheds light on their use as potential biomarkers and treatment targets for sepsis.

## Introduction

Sepsis is defined as a life-threatening organ dysfunction caused by a dysregulated host response to infection (Singer et al., 2016). According to clinical epidemiological studies, the estimated national incidence of sepsis and in-hospital mortality rate was approximately 5.9% and 15.6%, respectively, in the US, making it one of the major public health problems (Rhee et al., 2017). Notably, despite the development and improvement of clinical equipment and technology, the incidence as well as mortality due to sepsis are still high. According to the latest guideline of Surviving Sepsis Campaign, sepsis requires early diagnosis and treatment to improve prognosis and reduce death rate (Rhodes et al., 2017). However, biomarkers for its early identification as well as targeted therapies for sepsis are lacking. Therefore, it is important to actively explore the pathogenesis of sepsis. Moreover, sepsis could induce organ dysfunctions, such as cardiovascular dysfunction, acute lung injury (ALI), acute kidney injury (AKI), which in turn results in high mortality. Considering the dearth of knowledge regarding the pathophysiology of sepsis-induced organ dysfunction, is a need to carry out more further studies are required to elucidate the mechanisms involved (Lelubre and Vincent, 2018).

Studies on gene regulatory networks have focused on protein-coding genes. However, genomic analyses have determined that approximately 90% of noncoding sequences in the human genome are transcribed into noncoding RNAs (ncRNAs), which play a key regulatory role in multiple biological processes (Cech and Steitz, 2014). These ncRNAs are classified into two main subgroups based on their length: short ncRNAs (<200 nucleotides) and long ncRNAs (lncRNAs; >200 nucleotides) (Kapranov et al., 2007). Short ncRNAs mainly include miRNAs, and numerous studies have reported their involvement of in various diseases including sepsis (Mirna et al., 2019). However, unlike the extensively studied miRNAs, research on lncRNAs, which constitute majority of the noncoding transcriptome, is still in its infancy, though receiving increasing attention. Recent investigations have demonstrated that lncRNAs serve as master mediators of a wide range of biological processes and diseases such as cancer (Shang et al., 2020), cardiovascular diseases (Tang et al., 2020) and inflammatory disease (Peng et al., 2020). Notably, several investigations suggest that lncRNAs also participate in the pathological process of sepsis. Additionally, some studies have suggested that lncRNAs can be used as potential biomarkers. For instance, circulating lncRNA ZFAS1 presented good diagnostic and predictive values in sepsis that negatively correlated with disease risk and severity (Xu and Shao, 2019). Further, several recent studies have also confirmed that lncRNAs are involved in important regulatory functions in sepsis-induced organ dysfunctions; lncRNA PVT1 plays an important role in sepsis-induced heart dysfunction by regulating cell apoptosis in cardiomyocytes (Zhang et al., 2019). Thus, these reports on lncRNAs have revealed a new field of diagnostic and therapeutic opportunities in sepsis.

The current review aimed to evaluate the latest research progress with respect to the emerging roles of lncRNAs in sepsis. The basic overview of lncRNAs, their roles in sepsis and sepsis-induced organ dysfunctions, and the limitations of the current research are explored here.

## Overview of lncRNA

LncRNAs play essential roles in regulation of gene expression, despite not encoding proteins themselves (Kapranov et al., 2007). It is estimated that the number of lncRNA genes in mammals range from less than 20,000 to over 100,000 in humans (Kopp and Mendell, 2018). Depending on their genomic position relative to nearby protein-coding genes, lncRNA can be categorized as intergenic, intronic, bidirectional, sense, antisense, and enhancer lncRNA (Yang et al., 2019). According to their regulatory patterns, lncRNAs can be further classified into those that act *in cis*, influencing the expression and/or chromatin state of nearby genes, and those that execute an array of functions throughout the cell *in trans* (Kopp and Mendell, 2018). Interestingly, some lncRNAs were found to encode small peptides recently, thus escalating their complexity (Huang et al., 2017; Rossi et al., 2019).

**Figure 1:**
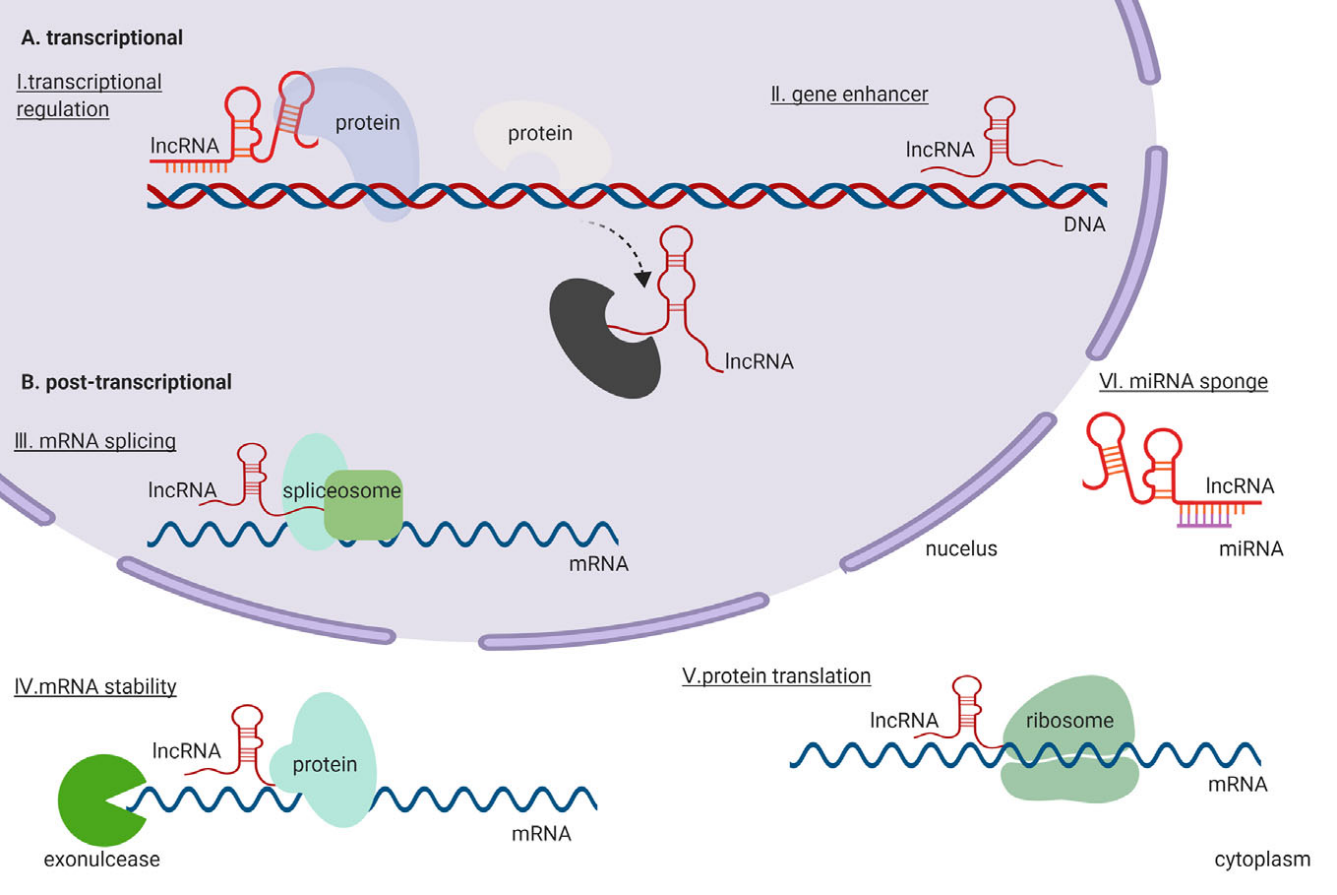
General functions and regulatory mechanisms of lncRNAs in the cell. **(A)** lncRNAs exert their functions in the transcriptional regulation. **(B)** lncRNAs exert their functions in the post-transcriptional regulation.

At the molecular level, lncRNAs exert their functions by directly binding to DNA, RNA, and proteins participating in the transcriptional and post-transcriptional regulation (**Figure 1**). During transcriptional regulation of target genes, lncRNAs repress or activate genes by functioning as either scaffold or decoy (Sun et al., 2016; Wu et al., 2019). Further, lncRNAs can function as enhancers for gene transcription (Pefanis et al., 2015; Huang et al., 2018). They can also co-transcriptionally regulate mRNA transcripts by altering their splicing (Yap et al., 2018). At the post-transcriptional level, lncRNAs can affect the stability of mRNA transcripts (Wang et al., 2019) and modulate mRNA translation by binding to ribosomes or mRNA transcripts during translation (Dimartino et al., 2018). Moreover, lncRNAs function as miRNAs sponge, which indirectly de-represses the expression of an mRNA that would be targeted by the miRNAs (Zhan et al., 2018). Thus, evidence indicates that lncRNAs are a group of large, diverse, and important transcripts that participate in regulation of gene expression through a variety of mechanisms.

## Role of lncRNAs in Sepsis

### lncRNAs as Potential Biomarkers for Sepsis

Novel biomarkers for sepsis are required because the existing inflammatory biomarkers for its diagnosis, such as C reactive protein (CRP), procalcitonin (PCT), and interleukin (IL)-6 (Enguix-Armada et al., 2016), have respective shortcomings in identification of sepsis onset. For example, PCT expression is altered in patients with severe trauma, pancreatitis, and cardiopulmonary resuscitation (Oppert et al., 2002; Sager et al., 2017; Parli et al., 2018). Additionally, CRP and IL-6 possess limited abilities to distinguish sepsis from other inflammatory conditions or to determine prognosis (Pierrakos and Vincent, 2010). Therefore, novel and more sensitive biomarkers are needed for early diagnosis of sepsis so as to improve prognosis in these patients. The recent development in high-throughput sequencing technology and gene chips, has enabled increasing number of studies to explore the expression patterns of lncRNAs in different diseases; studies demonstrated differential expression patterns of lncRNA under physiological and pathological conditions, thus confirming that lncRNAs may play important roles in various diseases including sepsis (Lu and Thum, 2019). For instance, Dai et al. studied lncRNA expression profiles using peripheral blood from three patients with sepsis and three healthy volunteers using microarray screening. They identified 1,316 differentially expressed (DE) lncRNAs between the two groups, and finally confirmed three lncRNAs, ENST00000452391.1, uc001vji.1, and uc021zxw.1, with significant differential expression. Interestingly, they also showed significant differential expression of lncRNAs ENST00000504301.1 and ENST00000452391.1 between sepsis survivors and non-survivors, illustrating that these lncRNAs may be good candidate biomarkers for sepsis (Dai et al., 2017). Besides, Cheng et al. performed an in silico investigation of the gene coexpression pattern for whole blood transcriptome of the patients response to all-cause sepsis in consecutive intensive care unit admissions. They identified five sepsis-related lncRNAs, including FENDRR, MALAT1, TUG1, CRNDE, and ANCR, whose functions were highly related with biological processes of sepsis (Cheng et al., 2020). Those studies demonstrated the involvement of lncRNAs in sepsis development and their potential as biomarkers in clinic.

Several studies suggested the potential of other lncRNAs, such as NEAT1 and MALAT1, as biomarker for sepsis (**Table 1**). NEAT1 was reportedly up-regulated in peripheral blood mononuclear cells (PBMCs) of patients with sepsis, with high sensitivity (67.85%) and specificity (87.27%) for sepsis diagnosis; however, no statistically significant relevance was observed between the sepsis survivors and non-survivors (Huang et al., 2017). Further, based on a study in 152 patients with sepsis and 150 healthy individuals, Huang et al. reported that the circulating lncRNA NEAT1 had better predictive value that positively correlated with sepsis severity, and thus proving its prognostic value (Huang et al., 2018). The difference in the prognostic value in these two studies may be due to different clinical samples and small size in each group. Moreover, He et al. corroborated the conclusions of Huang et al. and demonstrated that circulating lncRNA NEAT1/miR-124 axis had a better potency for diagnosis and prognosis of sepsis (He et al., 2019). These studies indicate that lncRNA NEAT1 may be an adaptive biomarker for sepsis.

**Table 1:** lncRNAs as potential biomarker for sepsis and sepsis-induced organ dysfunctions.

lncRNA	Sepsis patients/healthy controls	Diagnosis value of sepsis (AUC)	Prognostic value of mortality (AUC)	sample	level	Ref
lncRNA ZFAS1	202/200	0.814	0.628	plasma	down	(Xu and Shao, 2019)
lncRNA NEAT1	59/56	0.851	-	peripheral blood mononuclear cells	up	(Huang et al., 2017)
lncRNA NEAT1	152/150	0.730	0.641	plasma	up	(Huang et al., 2018)
lncRNA NEAT1/miR-124 axis	82/82	0.829	0.830	plasma	up	(He et al., 2019)
lncRNA MALAT1	120/60	0.910	0.886	plasma	up	(Chen et al., 2020)
lncRNA MALAT1	190/190	0.823	0.755	plasma	up	(Geng et al., 2019)
lncRNA MALAT1/miR-125a	196/196	0.931	0.678	plasma	up	(Liu et al., 2020)
lncRNA ITSN1-2	309/300	0.777	0.654	plasma	up	(Zeng et al., 2019)
lnc-MEG3/miR-21 axis	219/219	0.934	0.669	plasma	up	(Na et al., 2020)
lncRNA ANRIL	126/125	0.843	0.785	plasma	up	(Gui et al., 2019)
**lncRNA**	**Sepsis patients**	**Diagnosis value of organ dysfunctions (AUC)**	**Prognostic value of mortality (AUC)**	**sample**	**level**	**Ref**
		**Lung**				
lncRNA MALAT1	152	0.674	0.651	plasma	up	(Huang and Zhao, 2019)
Lnc-THRIL	109	0.706	0.780	plasma	up	(Wang et al., 2019)
		**Kidney**				
lncRNA TCONS_00016233	192	0.894	-	plasma	up	(Zhang et al., 2020)

Furthermore, several studies suggested that lncRNA MALAT1 could also participate in the process of sepsis and may be a potential biomarker for sepsis. Huang et al. demonstrated elevated lncRNA MALAT1 in sepsis non-survivors compared to that in survivors, with area under the curve (AUC) of 0.651 (Huang and Zhao, 2019). In addition, the studies performed by Chen et al. and Geng et al. showed circulating lncRNA MALAT1 might serve as a biomarker for sepsis with an AUC of 0.910 and 0.823, respectively, and prognosis with an AUC of 0.886 and 0.755, respectively (Geng et al., 2019; Chen et al., 2020). Liu et al. further demonstrated that lncRNA MALAT1/miR-125a axis also presented better diagnostic and prognostic values (Liu et al., 2020).

Furthermore, several studies demonstrated the biomarker potential of other lncRNAs, such as lncRNA ITSN1-2, lnc-MEG3, and lncRNA ANRIL. These three lncRNAs presented better diagnostic and predictive value for mortality, and positively correlated with the severity of sepsis (Gui et al., 2019; Zeng et al., 2019; Na et al., 2020). Moreover, lncRNA THRIL positively correlated with sepsis severity and also presented a good predictive value (AUC: 0.780) for mortality in patients with sepsis (Wang et al., 2019). However, lncRNA ZFAS1 negatively correlated with the severity of sepsis (Xu and Shao, 2019). These studies proved that lncRNAs could be considered as good additive markers for the diagnosis and prognosis of sepsis.

### Regulatory Mechanisms of lncRNAs in Sepsis

**Figure 2:**
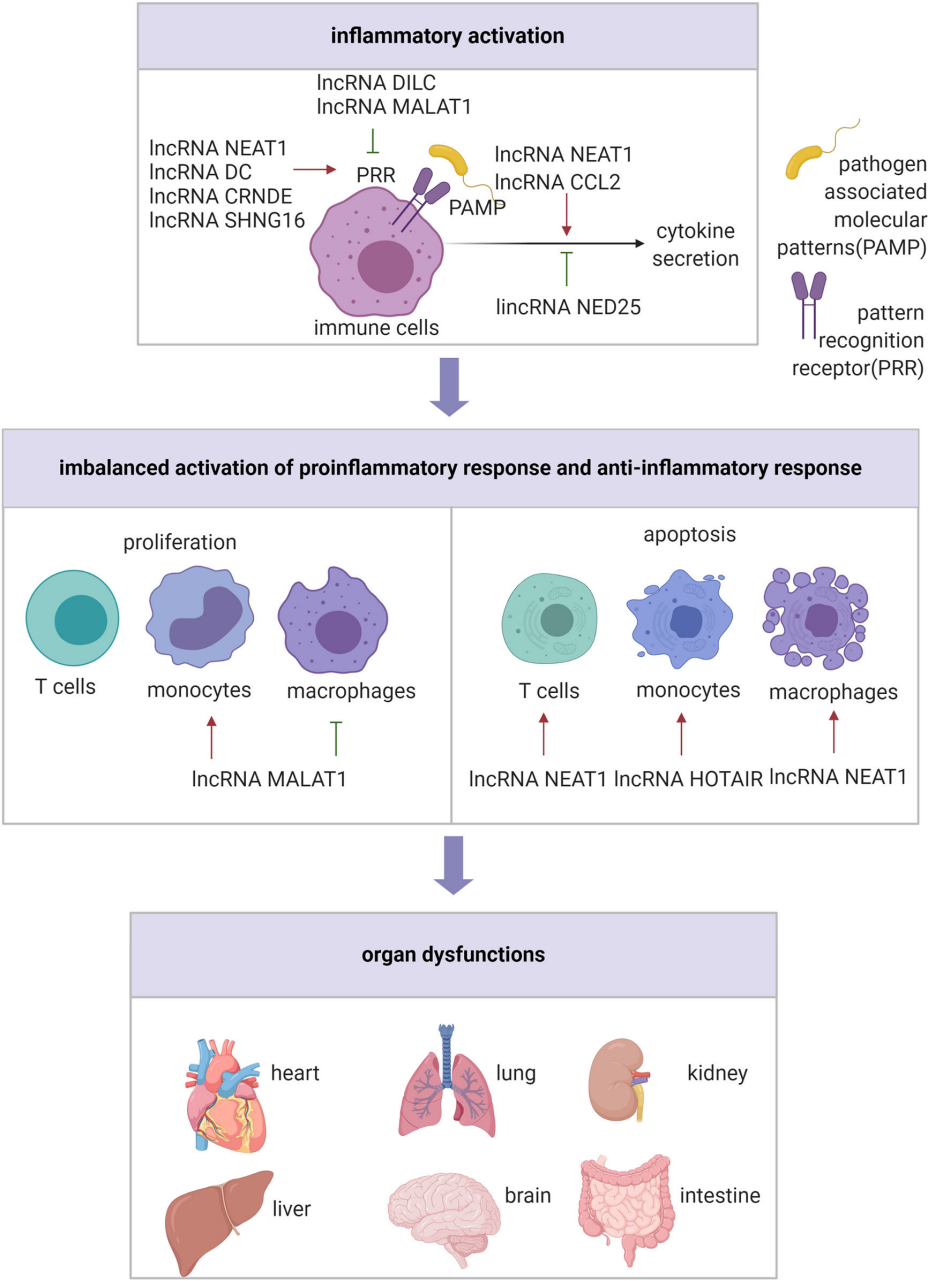
The regulatory role of lncRNAs in inflammatory state.

Sepsis is a disorder of the host response to infection, and the pro-inflammatory state coexists with immunosuppression (Bosmann and Ward, 2013; Singer et al., 2016) (**Figure 2**). Functional annotation analysis of lncRNA expression profiles in lipopolysaccharide (LPS)-induced human PBMCs indicated that the DE genes were primarily enriched in host immune and inflammatory response (Zhang et al., 2019). Notably, LPS could react with toll like receptor 4 (TLR4), inducing phagocytic cells to generate a variety of proinflammatory cytokines, such as IL-1β, tumor necrosis factor α (TNF-α), and IL-6, which is suggested to be a key pathway in sepsis pathophysiology (Bosmann and Ward, 2013). It has been reported that several lncRNAs are involved in the development of hyperinflammatory state in sepsis through the TLR4 signaling pathway. For instance, the expression of lncRNA NEAT1 was up-regulated in patient serum and associated with severity of sepsis. Silencing lncRNA NEAT1 suppressed LPS-induced inflammatory response in macrophages by mediating miR-17-5p and TLR4 (Li et al., 2020). Another lncRNA that participates in sepsis *via* TLR4 signaling pathway is acidic protein/four-disulfide core domain 21 (Wfdc21), also known as lncRNA DC, whose expression level was elevated in the cecal ligation and perforation (CLP)-induced animal model as well as LPS-induced macrophages. At the molecular level, the downregulation of Wfdc21 modulated the concentration of pro-inflammatory factors, mediated through the Stat3/TLR4 signaling pathway, in LPS-induced macrophages (Xie et al., 2018). Similarly, a study conducted by Wang et al. indicated that up-regulated lncRNA CRNDE and down-regulated miR-181a-5p were associated with shortened lifespan of patients with sepsis. Mechanistically, si-CRNDE-1 and miR-181a-5p mimic were able to reduce NF-κB, TNF-α, IL-1β, and IL-6 expression in LPS-induced mononuclear cells by targeting TLR4 through competitive endogenous RNA (ceRNA) mechanism (Wang et al., 2020). Additionally, Huang et al. demonstrated that lncRNA DILC could directly inhibit the expression of IL-6, which subsequently modulated TLR4-dependent inflammatory responses *via* Stat3 in LPS-induced mononuclear cells (Huang et al., 2018).

In addition to the TLR4 pathway, lncRNAs can affect the hyperinflammatory state in sepsis *via* other pathways. For example, high-level of lncRNA NEAT1 and low-level of miR-495-3p/miR-211 were associated with poor survival of mice with trauma. Mechanistically, miR-495-3p and miR-211 were sponged by lncRNA NEAT1, which possibly affected inflammation responses in LPS-induced macrophages by modulating Stat3 and PI3K/Akt signaling pathway, respectively (Xia et al., 2020). Moreover, it was found that elevation of proinflammatory cytokines caused by burn injury or post–burn infection was attenuated by treatment with lncRNA MALAT1 *via* the miR-214/TLR5 axis, thus providing a better understanding of the pathological regulation of post-burn sepsis (Gao et al., 2019). Furthermore, downregulation of lincRNA NED25 in PBMCs was associated with sepsis in patients, through modulation of miR-125b/Stat3/PCT/NO signaling pathway (Le et al., 2018). SIRT1, a highly conserved mammalian NAD^+^-dependent histone deacetylase, is associated with the regulation of immune function, and SIRT1 knockout mice are highly susceptible to sepsis (Gao et al., 2015). Jia et al. reported that the molecular mechanism for macrophage immune responses modulated by SIRT1 involved inhibition of lncRNA CCL2 expression by maintaining a repressive chromatin state in its locus, thereby reducing the inflammatory response in LPS-induced sepsis mice and macrophages (Jia et al., 2018). These findings indicate that lncRNAs are involved in the proinflammatory response of sepsis, and may be potential targets for its treatment.

The onset of sepsis in humans is accompanied by defective innate and adaptive immune response that can result in immunosuppression early in the course of sepsis, which is often long-lasting, impairing effective removal of infectious organisms and increasing susceptibility to secondary infection. Extensive apoptotic depletion of T cells, compromised innate immune functions of phagocytic cells, macrophage and dendritic cell dysfunction and depletion, which reportedly have lncRNAs involvement, contribute to immunosuppression in sepsis (Hotchkiss et al., 2009; Bosmann and Ward, 2013). Chen et al. observed that lncRNA NEAT1 could function as a ceRNA for miR-125 to upregulate MCEMP1, which increased inflammatory cytokines in serum, promoted apoptosis and inhibited T lymphocyte viability in septic mice (Chen et al., 2019). lncRNA HOTAIR promoted the progression of sepsis, by inhibiting monocyte proliferation, while promoting monocyte apoptosis and inflammation response; this was achieved by acting as miR-211 sponge, thereby inducing IL-6R expression in CLP-induced sepsis mice model (Chen et al., 2019). lncRNA MALAT1 also promoted inflammation and proliferation of monocytes, while inhibiting monocyte apoptosis in CLP-induced septic mice; these effects, at the molecular level, were mediated through the binding of lncRNA MALAT1 to miR-23a and up-regulated MCEMP1 through loss- and gain-of-function experiments *in vivo* (Xie et al., 2020). Another study confirmed that lncRNA NEAT1 promoted the inflammation and apoptosis while restraining the proliferation of LPS-induced macrophages *via* the miR-370-3p/TSP-1 axis (Wu et al., 2020). Further, Yang et al. showed that lncRNA MALAT1 was down-regulation in 50 patients with sepsis compared to 50 controls; this down-regulation inhibited macrophage proliferation by inhibiting has-miR-346 targeting SMAD3 in LPS-induced mouse macrophages (Yang et al., 2019). These studies suggested that lncRNAs regulate immune cell apoptosis and proliferation, which provides a new perspective for immunosuppression in sepsis. Thus, lncRNAs may be potential therapeutic targets.

### lncRNAs in Children With Sepsis

Neonatal sepsis is a typical blood infection that is mainly caused by bacteria. Neonatal sepsis has become a huge challenge for neonatologists due to its high incidence and unclear pathogens (Shane et al., 2017). Recently, several studies found that lncRNAs could also play a role in the process of neonatal sepsis. Liu et al. established a ceRNA network identifying lncRNA HCP5, LINC00638, lncRNA XIST, and lncRNA TP53TG1 as hub nodes; functional analysis of this network identified essential immune functions, hematopoietic functions, osteoclast differentiation, and primary immunodeficiency associated with neonatal sepsis (Liu et al., 2020). In addition, Wang et al. reported that miR-15a/16 was up-regulated in serum of patients with neonatal sepsis; investigations into its mechanism revealed that lncRNA SHNG16 could act as a ceRNA and positively regulate TLR4 by competitively binding miR-15a/16 in LPS-induced macrophages, thus indicating that lncRNA SNHG16 affected inflammatory responses in neonatal sepsis (Wang et al., 2018). Apart from neonatal sepsis, Bai et al. reported a total of 1,488 DE lncRNAs and 1,460 DE mRNAs between ten pediatric patients with sepsis and twelve healthy controls; they conducted a co-expression network analysis of the DE lncRNAs and mRNAs, suggesting a correlation between lncRNA and mRNA expression that were involved in the pathogenesis of sepsis (Bai et al., 2020). Considering the different pathophysiological processes between children and adult sepsis (Weiss et al., 2020), further research is needed to elucidate the roles of lncRNAs and explore their molecular mechanisms in children with sepsis.

## Role of lncRNAs in Sepsis-Induced Organ Dysfunctions

**Figure 3:**
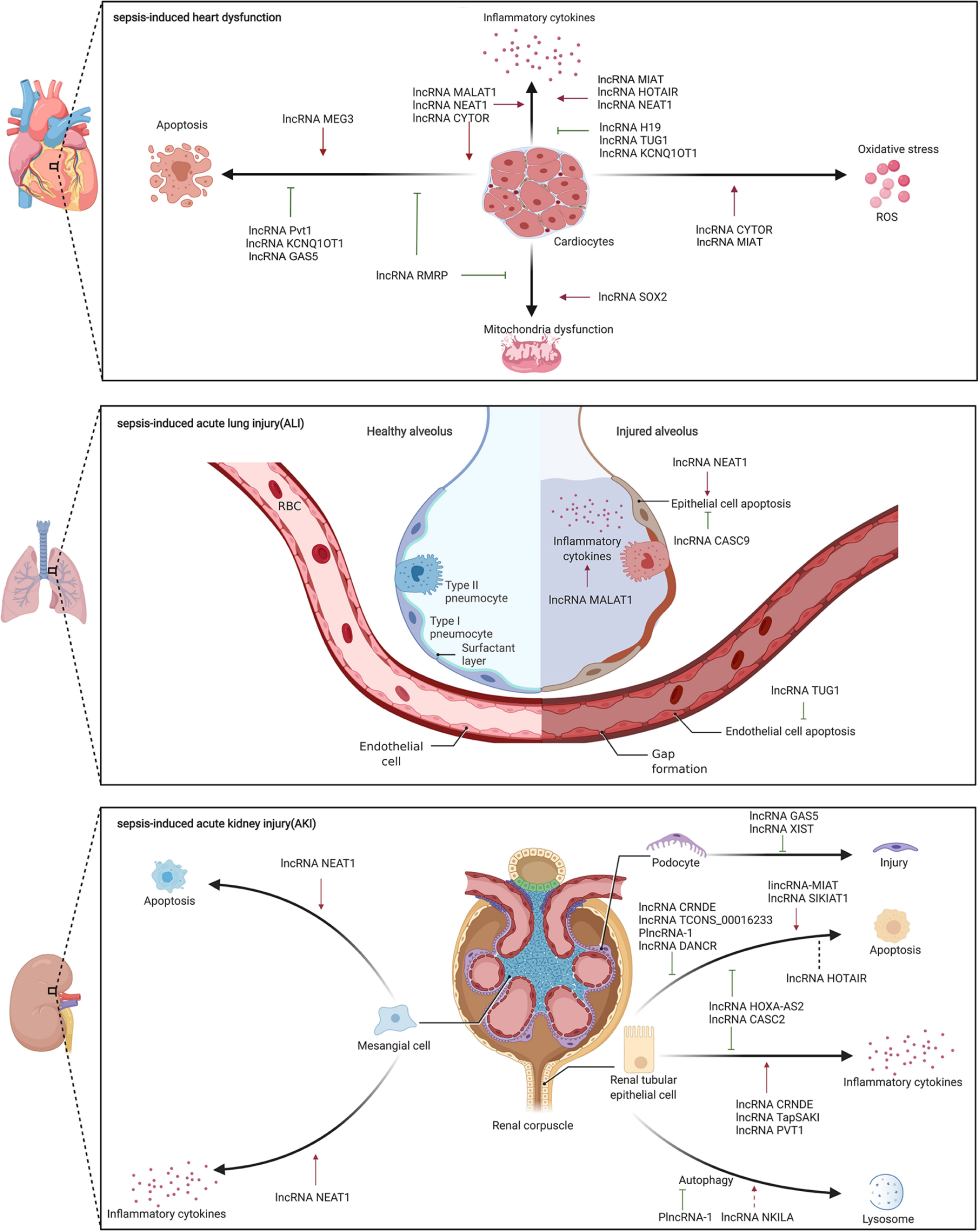
The role of lncRNA in sepsis-induced organ dysfunctions.

Sepsis is often accompanied by various organ dysfunctions, including cardiovascular dysfunction, ALI, AKI, which is one of the reasons for the high mortality of sepsis (Lelubre and Vincent, 2018). However, the mechanisms of sepsis-induced organ dysfunctions are still unclear. Elucidating the pathophysiology of organ dysfunctions in sepsis is crucial for optimizing the management and treatment of patients as well for the development of potential new therapies (Pool et al., 2018). Recent studies suggested that lncRNAs could regulate a variety of biological processes and are reportedly involved in the pathophysiology of sepsis-induced organ dysfunctions, thus highlighting their importance (**Figure 3**).

### Role of lncRNAs in Sepsis-Induced Cardiovascular Dysfunction

Sepsis-induced cardiovascular dysfunction could lead to an unacceptably high mortality rate. Patients with sepsis-induced heart dysfunction typically exhibit ventricular dilatation, reduced ventricular contractility, and/or both right and left ventricular dysfunction with a reduced response to volume infusion (Martin et al., 2019). In addition, sepsis-induced vascular dysfunction, especially endothelial dysfunction, can increase vascular permeability, promote activation of the coagulation cascade, cause tissue edema, and compromise perfusion of vital organs, which is a central event in the pathogenesis of sepsis (Bermejo-Martin et al., 2018). However, the basic pathophysiological mechanisms underlying sepsis-induced heart and vascular dysfunction have not yet been completely elucidated. Recent studies have shown that lncRNAs are involved in sepsis-induced heart and vascular dysfunction.

### Role of lncRNAs in Sepsis-Induced Heart Dysfunction

Our team showed 74 DE lncRNAs in left ventricular tissues of 6 sepsis rats and 6 control rats using RNA-Seq. Further bioinformatics analyses suggested that cell apoptosis and its related pathways were enriched, which indicated that cell apoptosis may play a critical role in the process of heart dysfunction. Further, we found that lncRNA Pvt1 knockdown increased cell apoptosis in H9c2 cell lines through upregulation of pro-apoptosis protein expression and down-regulation of anti-apoptosis protein expression; lncRNA Pvt1 overexpression reversed the results *in vitro* (Zhang et al., 2019). In addition, a study performed by Chen et al. indicated that high lncRNA MEG3 expression was associated with high mortality rates in patients with sepsis, and *in vitro* experiments revealed that lncRNA MEG3 increased LPS-induced cardiomyocyte and renal epithelial cell apoptosis (Chen et al., 2019). Another study conducted by Li et al. confirmed that lncRNA GAS5 was downregulated in sepsis patients and in LPS-induced AC16 cells. Overexpression of lncRNA GAS5 could suppressed LPS-induced cardiomyocytes apoptosis by regulating methylation of miR-124 *in vitro (* Li et al., 2020).

Mitochondria are the source of energy production, and their dysfunction directly affects the contractile function of myocardial cells; abnormal contractile function is an important cause of sepsis-induced heart dysfunction. Gain- and loss-of-function experiments in LPS-induced sepsis rats and H9c2 cell line revealed that lncRNA SOX2 could aggravate cardiac function and mitochondrial dysfunction by targeting SOX2 (Chen et al., 2019); therefore lncRNA could influence the of mitochondrial function and thus participate in the process of sepsis-induced heart dysfunction.

Excessive inflammatory response can exacerbate sepsis-induced heart dysfunction; several studies have shown lncRNA MALAT1 involvement *via* regulation of inflammatory response. Chen et al. showed that lncRNA MALAT1 was upregulated, aggravating cardiac inflammation and heart dysfunction in CLP sepsis rat model and LPS-induced H9c2 cells *via* interactions with miR-125b and p38 MAPK/NF-κB (Chen et al., 2018). Another study showed in the LPS-induced H9c2 cell line, lncRNA MALAT1 depletion, through its binding to miR-150-5p, decreased the inflammatory response in cells (Wei and Liu, 2019). Moreover, lncRNA HOTAIR also participated in sepsis-induced heart dysfunction by regulating the inflammatory response. At the molecular level, silencing lncRNA HOTAIR improved heart function in LPS-induced sepsis mice, and markedly decreased TNF-α production by activating NF-κB pathway, involving phosphorylation of NF-κB p65 subunit, in LPS-induced HL-1 cells (Wu et al., 2016). Similarly, Wang et al. reported that lncRNA NEAT1 knockdown improved heart function and alleviated myocardial injury in LPS-induced sepsis mice, possibly by inhibiting the TLR1/NF-κB signaling pathway (Wang et al., 2019). Furthermore, lncRNA H19 acted as ceRNA for AQP1 in miR-874 regulation, and restored LPS-induced inflammatory response and heart dysfunction *in vivo* and *in vitro* (Fang et al., 2018). Moreover, lncRNA TUG1 was downregulated in sepsis and might sponge miR-27a to downregulate expression of TNF-α, thereby inhibiting apoptosis of LPS-induced AC16 cells (Wang et al., 2020). These studies indicate that lncRNAs may participate in sepsis-induced heart dysfunction by regulating inflammation.

Some studies have reported that lncRNAs target and regulate various pathological processes in sepsis-induced heart dysfunction. Han et al. reported that overexpression of lncRNA RMRP in LPS-induced sepsis mice and HL-1 cells alleviated apoptosis and mitochondrial damage by acting as a sponge for miR-1-5p, which targets HSP70 protein 4 (Han et al., 2020). Zhuang et al. showed that lncRNA MALAT1 knockdown impaired TNF-α expression, by targeting serum amyloid antigen 3, and reduced LPS-induced HL-1 cell apoptosis *in vitro* (Zhuang et al., 2017). Similarly, lncRNA NEAT1 knockdown promoted cell viability, suppressed cell apoptosis, and alleviated inflammatory response in LPS-induced HL-1 cells by interacting with miR-144-3p *via* NF-κB pathway (Wei et al., 2020). Furthermore, Sun et al. showed that overexpression of lncRNA KCNQ1OT1 improved heart function in LPS-induced sepsis rat; lncRNA KCNQ1OT1 attenuated myocardial apoptosis and inflammatory factors by sponging miR-192-5p that targets X-chromosome-linked inhibitor of apoptosis (XIAP) in LPS-induced H9c2 cells (Sun et al., 2020). Chen et al. reported that down-regulation of lncRNA CYTOR, another lncRNA that regulates XIAP, exacerbated sepsis-induced heart dysfunction by promoting apoptosis and inflammatory response as well as oxidative stress *in vivo* and *in vitro* (Chen et al., 2020). Another study performed by Xing et al. suggested that lncRNA MIAT could promote inflammation response as well as oxidative stress in LPS-induced HL-1 cells. At the molecular level, lncRNA MIAT could target against miR-339-5p to activate TRAF6/NF-κB axis *in vitro (* Xing et al., 2020). These studies indicate that lncRNAs participate in sepsis-induced heart dysfunction by regulating multiple pathways; however, more studies are needed to verify their clinical value.

#### Role of lncRNAs in Sepsis-Induced Vascular Dysfunction

A systematic transcriptional survey performed by Singh et al. showed 871 significantly upregulated and 1,068 significantly downregulated lncRNAs in LPS-induced human umbilical vein endothelial cells compared to vehicle-treated controls (Singh et al., 2016). Similarly, another study on lncRNA expression profiles in human microvascular endothelial cells (HMECs) subjected to LPS treatment revealed that 2,426 and 8,355 lncRNAs that were either up- or downregulated, respectively, in LPS-treated HMECs at 3 h, and a total of 3,601 and 4,709 lncRNAs were either up- or down-regulated, respectively, after a 24-h treatment. The DE lncRNAs, including lncRNA EGO, lncRNA HOTAIRMI, lnc-IL7R, were further validated in LPS-induced HMECs (Chowdhury et al., 2017). These two studies showed that lncRNAs are involved in LPS-induced endothelial dysfunctions. Additionally, down-regulation of lncRNA UCA1 and lncRNA HULC in LPS-induced sepsis mice and HMECs alleviated vascular injury associated with reduced ICAM1, VCAM1, and IL-6 (Chen et al., 2019). Moreover, Yu et al. reported that ulinastatin, a protease inhibitor, significantly reduced LPS-induced cardiac microvascular endothelial cell permeability and the percentage of apoptotic cells by inhibiting lncRNAs MALAT1 and EZH2 (Yu et al., 2017). Furthermore, Chen et al. suggested that lncRNA HULC and TRPM7 were up-regulated in sepsis patients and in LPS-induced HUVECs, while miR-204-5p was down-regulated. At the molecular level, it was confirmed that lncRNA HULC/miR-204-5p/TRPM7 network could play a pivotal role in the process of apoptosis, inflammation and oxidative stress in LPS-induced HUVECs (Chen and Song, 2020). However, there are only a few studies so far on the involvement of lncRNAs in sepsis-induced vascular dysfunctions, which warrants further research.

### Role of lncRNAs in Sepsis-Induced ALI

The lung is one of the major organs susceptible to sepsis-induced dysfunction. Acute respiratory distress syndrome (ARDS), which is the clinical term for ALI, is one of the most critical prognostic factors for mortality in patients with sepsis (Lagu et al., 2012). Alveolar epithelial/endothelial cell injury and activation of innate immune response are pathological hallmarks of this clinical phenotype (Fanelli and Ranieri, 2015). Despite a thorough understanding of its pathophysiology, specific therapeutic targets need to be explored for better treatment. Notably, recent studies have reported the association of some lncRNAs with sepsis-induced ALI, thus providing a new theoretical basis for research in clinic.

To date, two clinical studies have reported lncRNAs related to sepsis-induced ALI (**Table 1**). A study on 152 patients with sepsis found increased lncRNA MALAT1 expression in patients with ARDS compared to those without ARDS (Huang and Zhao, 2019). Another study on 109 patients with sepsis reported upregulation of lncRNA THRIL in ARDS group compared with non‐ARDS group; moreover, it could effectively distinguish between patients with and without ARDS (AUC: 0.706) (Wang et al., 2019). These two studies illustrate the potential of lncRNAs to serve as diagnostic biomarkers for sepsis-induced ARDS. However, more clinical studies are needed to elucidate the relationship between lncRNAs and sepsis-induced ALI.

Experimental studies have found that several lncRNAs are involved in the physiological process of sepsis-induced ALI. Injury to epithelial cells, key component of the alveolar barrier, aggravates sepsis-induced ALI. Along these lines, Zhou et al. found that lncRNA NEAT1 might aggravate the progression of ALI by inducing alveolar epithelial cell apoptosis and inflammation *via* the high-mobility group box1/receptors for advanced glycation end products/NF-κB signaling (Zhou et al., 2020). Another study showed that lncRNA MALAT1 promoted inflammation in LPS-induced alveolar epithelial cells by targeting the MyD88/NF-κB pathway but lacked *in vivo* evidence (Liang et al., 2019). Additionally, it was also reported found that lncRNA CASC9 protected alveolar epithelial cells from apoptosis by regulating miR-195-5p/pyruvate dehydrogenase kinase axis (Wang et al., 2020). Pulmonary endothelial cell injury is another key factor in sepsis-induced ALI. Qiu et al. showed that lncRNA TUG1 alleviated sepsis-induced ALI by reducing endothelial cell apoptosis and inflammation by targeting miR-34b-5p and GRB2-associated binding protein 1 (Qiu et al., 2020). These lncRNAs, reportedly involved in sepsis-induced ALI, are still limited in number and need to be further verified by the animal and clinical studies.

### Role of lncRNAs in Sepsis-Induced AKI

Sepsis can lead to renal ischemia, decreased renal perfusion, and renal dysfunction, which is known as AKI. Although AKI is one of the most important causes of death in patients with sepsis (Poston and Koyner, 2019), the pathogenesis of sepsis-induced AKI is still unclear. Emerging evidence indicates the importance of lncRNAs in the occurrence and development of sepsis-induced AKI. Chun et al. reported an analysis of circulating lncRNA expression profiles in control and septic AKI patients using lncRNA microarrays, with 5,361 up-regulated and 5,928 down-regulated lncRNAs (Chun-Mei et al., 2016). Additionally, three lncRNAs, MIR210HG, linc-ATP13A4-8, and linc-KIAA1737-2, were up-regulated in human proximal tubular epithelial cells exposed to human septic AKI plasma as compared to those exposed to septic non-AKI or control plasma (Lin et al., 2015). Both studies illustrated the possible involvement of lncRNAs in the pathogenesis of sepsis-induced AKI.

Two lncRNAs were reported to be potential biomarkers for the early diagnosis of sepsis-induced AKI (**Table 1**). Zhang et al. evaluated the expression profile of circulating lncRNA in septic AKI patients (n = 15), septic non-AKI (n = 15) patients, and healthy controls (n = 15); based on lncRNA chip assay, they identified lncRNA TCONS_00016233 as the most up-regulated lncRNA with a sensitivity and specificity of 71.9% and 89.6%, respectively for the detection of AKI (Zhang et al., 2020). Additionally, lncRNA NEAT1 was also elevated in the serum of patients with sepsis-induced AKI compared with controls and positively correlated with the severity of AKI (Chen et al., 2018). These studies indicate that lncRNAs may be able to detect sepsis-induced AKI early in its course and thus facilitate early treatment.

Studies in animal models indicated that lncRNAs may be involved in specific pathophysiological processes of sepsis-induced AKI, such as apoptosis, autophagy, and inflammatory response. Recent reports suggested that several lncRNAs regulate apoptosis mainly through ceRNA mechanism in sepsis-induced AKI. A remarkable increase in lncRNA MIAT was detected in LPS-induced sepsis rats and rat kidney epithelial cells; further, lncRNA MIAT regulated the expression of the apoptosis protein caspase8 by binding to miR-29a *in vitro* (Zhang et al., 2019). Additionally, lncRNA HOXA-AS2 expression was diminished in patients with sepsis, CLP-induced sepsis mice, and LPS-induced HK-2 cells; *in vitro* studies found that overexpression lncRNA HOXA-AS2 reduced cell apoptosis and inflammation by targeting miR‐106b‐5p and hindering the Wnt/β‐catenin and NF‐κB pathway (Wu et al., 2020). Moreover, overexpression lncRNA CRNDE in rats ameliorated sepsis-induced AKI. Mechanistically, lncRNA CRNDE sponged miR-181a-5p targeting PPAR-α to promote cell proliferation and inhibit cell apoptosis through loss- and gain-of-function experiments *in vitro* (Wang et al., 2020). Similarly, lncRNA TCONS_00016233 ameliorated kidney injury in LPS- and CLP-induced rat AKI models by regulating apoptosis. At the molecular level, lncRNA TCONS_00016233 could act as a ceRNA to prevent miR-22-3p-mediated downregulation of the AIFM1 *in vivo* and *in vitro* (Zhang et al., 2020). Interestingly, two studies have reported contradictory effects of lncRNA HOTAIR. Jiang et al. reported that overexpression lncRNA HOTAIR could alleviate kidney injury in sepsis rats by inhibiting apoptosis of kidney tissues through downregulation of the miR-34a/Bcl-2 signaling pathway (Jiang et al., 2019). Whereas, Shen et al. showed that lncRNA HOTAIR knockdown relieved kidney injury in urine-derived sepsis rat model; lncRNA HOTAIR promoted cell apoptosis *via* miR-22/HMGB1 pathway *in vivo* and *in vitro* (Shen et al., 2018). These contradictory results may be because of the difference in animal models studied. Nonetheless, it is clear that lncRNAs play a role in sepsis-induced AKI by regulating apoptosis.

As mentioned previously, besides apoptosis, lncRNAs may be involved in the regulation of sepsis-induced AKI through other mechanisms such as autophagy and inflammatory response. Inflammatory injury plays an important role in sepsis-induced AKI, and NF-κB signaling pathway that is vital in regulating inflammatory response can be regulated by lncRNAs. Sun et al. found that lncRNA CRNDE induced sepsis-induced AKI by activating the TLR3/NF-κB signaling pathway, which could be inhibited by lncRNA CRNDE knockout *in vivo* (Sun et al., 2019). Similarly, knockdown lncRNA TapSAKI alleviated kidney injury in urine-derived sepsis rat model by targeting the miR-22/PTEN/TLR4/NF-κB signaling pathway through ceRNA mechanism (Shen et al., 2019). Further, involvement of lncRNAs in the pathology of sepsis-induced AKI was suggested by Yang et al., who reported that up-regulated lncRNA NKILA might be involved in regulation of autophagy through Akt pathway in LPS-induced HK-2 cells (Yang et al., 2019). These *in vivo* studies confirmed that lncRNAs are involved in sepsis-induced AKI through regulation of apoptosis, autophagy, and inflammatory responses, and are thus expected to serve as biomarkers and new therapeutic targets.

lncRNA NEAT1 suppression reduced cell apoptosis, inflammation response, and oxidative stress in LPS-induced rat mesangial cells by regulating miR-204/IL-6R and NF-κB pathways (Chen et al., 2018). Fu et al. found that PlncRNA-1 was downregulated in the serum of patients with septic AKI and in LPS-induced NRK-52E cells, a renal tubular epithelial cell line. Further *in vitro* research found that PlncRNA-1 overexpression promoted cell proliferation and inhibited apoptosis and autophagy by regulating BCL2 expression (Fu et al., 2018). Similarly, in LPS-induced HK-2 cells, another renal tubular epithelial cell line, gain- and loss-of-function experiments revealed that lncRNA PVT1 promoted inflammatory response by binding to TNF-α and regulating JNK/NF-κB signaling pathway (Huang et al., 2017). Moreover, Zhao et al. reported that lncRNA DANCR was down-regulated in serum of patients with sepsis-induced AKI and LPS-induced HK-2 cells; whereas, its overexpression reduced apoptosis and increased cell viability by sponging miR-214 and inhibiting Krüppel-like factor-6 *in vitro* (Zhao et al., 2020). Furthermore, lncRNA CASC2 promoted cell viability and inhibited secretion of inflammatory factors, apoptosis, and oxidative stress in LPS-induced HK–2 cells by inhibiting miR–155 and NF–κB pathway (Wang et al., 2020). Lu et al. showed that lncRNA ENST00000452391.1, also called lncRNA SIKIAT1, acted as a ceRNA for miR-96-3p and enhanced FOXA1 expression and promoted HK-2 cell apoptosis (Lu et al., 2020). In LPS-induced podocytes, lncRNA GAS5 expression was decreased, and its knockdown markedly enhanced podocyte injury by promoting PI3K/Akt pathway through PTEN (Fang et al., 2018). Additionally, lncRNA XIST reduced LPS-induced mouse podocyte apoptosis by sponging miR-15a-5p and targeting CUL3 (Xu et al., 2019). These studies indicate that lncRNAs play important regulatory roles in sepsis-induced AKI cell models, including mesangial cells, renal tubular epithelial cells, and podocytes, which are important components of the kidney. However, these roles of lncRNAs need further verification through animal and clinical studies.

### Role of lncRNAs in Sepsis-Induced Other Organ Dysfunctions

In addition to sepsis-induced cardiovascular dysfunction, ALI, and AKI, sepsis can also lead to liver injury, brain injury, skeletal muscle dysfunction, and intestinal dysfunction (Gotts and Matthay, 2016). Recent studies have shown that several lncRNAs participate in their pathophysiological processes and can thus be used as potential new therapeutic targets.

The liver, which performs vital functions such as metabolism and detoxification, is vulnerable to sepsis (Yan et al., 2014). Recently, two lncRNAs were reported to be involved in sepsis-induced liver injury. Zhang et al. found that lncRNA NEAT1 was upregulated in patients with sepsis-induced liver injury. Functional studies in LPS-induced macrophages showed that lncRNA NEAT1 interacted with Let-7a, targeting TLR4 that in turn contributed to the LPS-induced inflammatory response (Zhang and Niu, 2019). Another lncRNA, which functions *via* ceRNA mechanism, lncRNA CRNDE overexpression in rats ameliorated sepsis-induced liver injury by inhibiting hepatic epithelial cell apoptosis through binding to miR-126-5p, and in turn promoting the expression of BCL2L2 (Li et al., 2020).

Sepsis-induced brain injury is related to cognitive sequelae in patients admitted to the intensive care unit and can have serious impact on quality of life after recovery (Kuperberg and Wadgaonkar, 2017). Sun et al. explored a high throughput transcriptome sequencing in rat brain tissue of group exposed to LPS for 6 h (n=20), group exposed to LPS for 24 h (n=20), and group without LPS (n=20); they reported increased expression of 316 lncRNAs and decreased expression of 84 lncRNAs after 6 h LPS exposure, and increased expression of 117 lncRNAs and decreased expression of 79 lncRNAs after 24 h exposure. Bioinformatics analyses found that microtubule malformation and dysfunction might be involved in the pathogenesis of sepsis-induced brain injury (Sun et al., 2017). In addition, lncRNA Lethe knockout in CLP-induced sepsis mice aggravated sepsis-induced brain injury by decreasing autophagy (Mai et al., 2019). On the other hand, lncRNA NEAT1 knockdown in CLP-induced sepsis mice alleviated brain injury by reducing apoptosis and downregulating NF-κB pathway (Liu et al., 2019). These three studies illustrate that lncRNAs are closely related to sepsis-induced brain injury.

Sepsis can reduce the force production capacity of muscles as well as skeletal muscle mobility, thereby inducing muscle atrophy and serious skeletal muscle injury. Recently, Yong et al. reported that lncRNA MALAT1, through interaction with EZH2, promoted Akt-1 phosphorylation, decreased BRCA1 expression, and exported BRCA1 from the nucleus, thus promoting skeletal muscle cell apoptosis and inflammatory responses, and ultimately accelerating the progression of sepsis *in vivo* and *in vitro* (Yong et al., 2020). Furthermore, in sepsis-induced intestinal barrier dysfunction, Yu et al. reported that lncRNA H19 was upregulated in CLP-induced intestinal mucosal tissues in sepsis mice and patients with sepsis. At the molecular level, targeted deletion of lncRNA H19 in CLP-induced sepsis mice enhanced the function of Paneth and goblet cells and promoted autophagy (Yu et al., 2019).

Thus, lncRNAs are vital in sepsis-induced organ dysfunctions but are seldom studied. Further research to explore the role of other lncRNAs in sepsis-induced organ dysfunctions and clinical tests to confirm their importance are needed in the future.

## Conclusion and Perspectives

RNA-sequencing has provided an unprecedented insight into the human genome. The field of lncRNA is growing at a blistering pace with several labs investigating various diseases including sepsis. As detailed in this review, lncRNAs participate in the pathology of sepsis and sepsis-induced organ dysfunctions, and can serve as potential biomarkers and therapeutic targets. However, research on lncRNAs in sepsis is far from complete. Sepsis is involved in not only early activation of pro- and anti-inflammatory responses, but also major modifications in non-immunologic pathways such as cardiovascular, neuronal, autonomic, hormonal, bioenergetic, metabolic, and coagulation, all of which may be regulated by lncRNAs (Singer et al., 2016). Therefore, further research is needed to study the roles of lncRNAs in these pathological processes. In addition, clinical trials to verify the biomarker and therapeutic potential of lncRNAs in human sepsis are lacking and are required in the future. As there is no unified biomarker for sepsis-induced organ dysfunctions at present, research devoted to this area is needed to explore better biomarkers including lncRNAs in the future. Gaining a better understanding of the regulatory roles of lncRNAs in sepsis will provide novel insights into the molecular mechanisms governing sepsis. Thus, this review systematically highlighted the recent advances with respect to the role of lncRNAs in sepsis, which may aid the design of future experimental research, and thereby reveal the diagnostic and therapeutic potential of lncRNAs in sepsis.

## Author Contributions

WW, NY, RW, C-FL, and T-NZ wrote the review. All authors contributed to the article and approved the submitted version.

## Funding

This work was supported by the National Natural Science Foundation of China (81372039 and 81971810) and the Liaoning Providence Science and Technology project (2020JH1/10300001).

## Conflict of Interest

The authors declare that the research was conducted in the absence of any commercial or financial relationships that could be construed as a potential conflict of interest.
